# Using cognitive modeling to examine the effects of competition on strategy and effort in races and tournaments

**DOI:** 10.3758/s13423-022-02213-x

**Published:** 2022-11-16

**Authors:** Andrew J. Morgan, Andrew Neal, Timothy Ballard

**Affiliations:** School of Psychology, University of Queensland, Brisbane, Australia

**Keywords:** Computational modeling, Decision making

## Abstract

We investigated the effects of two types of competition, races and tournaments (as well as an individual challenge and a do-your-best condition), on two different aspects of performance: effort and strategy. In our experiment, 100 undergraduate participants completed a simple cognitive task under four experimental conditions (in a repeated-measures design) based on different types of competitions and challenges. We used the Linear Ballistic Accumulator to quantify the effects of competition on strategy and effort. The results reveal that competition produced changes in strategy rather than effort, and that trait competitiveness had minimal impact on how people responded to competition. This suggests individuals are more likely to adjust their strategy in competitions, and the uncertainty created by different competition types influences the direction of these strategy adjustments.

**Supplementary Information:**

The online version contains supplementary material available at 10.3758/s13423-022-02213-x.

## Introduction

Competition occurs when multiple actors attempt to gain some resource that cannot be evenly shared (Deutsch, 1949). To compete against an opponent, we adjust our strategy and the effort we apply to maximize our opportunities for success. These adjustments are often made in the presence of uncertainty, the nature of which depends on the type of competition. In a race, the competitors strive to be the first to achieve a performance target, which creates uncertainty in the time each competitor needs to beat. In a tournament, the competitors strive to outperform each other within a specific time frame, which creates uncertainty in the performance level required to win. This uncertainty makes it difficult to know the best strategy to employ, and the effort required.

Surprisingly little is known about the ways people regulate strategy and effort when competing. Many studies have examined the effects of competition on performance; however, the findings have been inconsistent. Some studies have found that competition helps performance (Michaels, 1977; Scott & Cherrington, 1974), some have found that it hinders performance (Johnson et al., 1981; Slavin, 1977) while others found no overall effect (Murayama & Elliot, 2012). Progress has been made in explaining these inconsistencies by identifying the motivational and dispositional variables that predict the outcomes of competition, such as competitiveness and self-confidence (S. P. Brown et al., 1998; Tuckman, 2003). However, less is known about the underlying cognitive mechanisms.

Our aim is to examine the cognitive mechanisms by which different competition types influence performance. To do this, we had participants compete in tournaments and races against a simulated opponent and used cognitive modeling to quantify the latent processes underlying performance. We used a speeded choice task and fit the Linear Ballistic Accumulator (S. D. Brown & Heathcote, 2008) to participants’ data. We examined how the parameters of the model respond to different types of competition and assess whether these are affected by trait competitiveness.

### Effects of competition types on effort and strategy

Previous research, though not explicitly differentiating between races and tournaments, has looked at their impact on behavioral indicators of effort. Some studies operationalize competitions as a race (Chapsal & Vilain, 2019; Kilduff, 2014). For example, Zizzo (2002) examined participants racing to be the first to make 10 steps of “progress.” At each step in the race, participants invested points, with bigger investments giving greater chances of progress. They found tied competitors invested more when closer to the end of the race. Participants’ behavior therefore changed depending on goal proximity, and the individual’s place in the race. Other studies operationalized competition as a tournament (Casas-Arce and Martinez-Jerez, 2009; Haines & McKeachie, 1967). For example, Huang et al. (2017), examined a competition in which participants played a dice-rolling game, with the winner having the most points after a certain number of rounds. They assessed effort using measures of persistence (time spent on task). Huang et al. (2017) found being ahead in the competition increased persistence in early stages but decreased persistence in later stages. These inconsistencies in how competition has been operationalized and effort has been measured make drawing conclusions difficult.

**Fig. 1:**
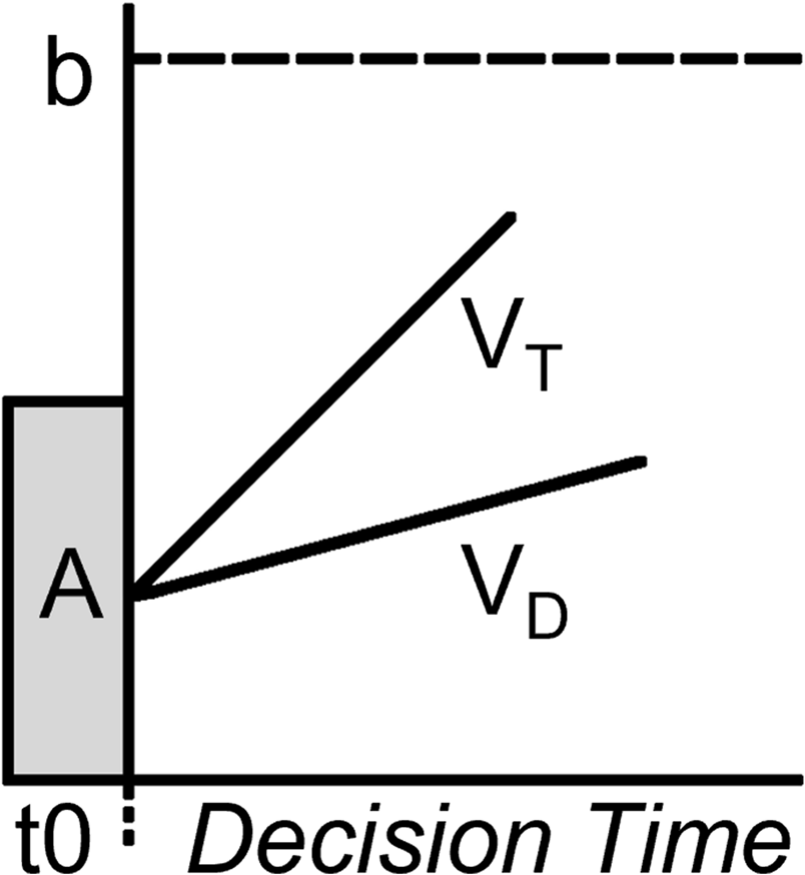
The Linear Ballistic Accumulator model. The b parameter represents the threshold for decision-making and the V parameters represent the rate of evidence accumulation. Once enough evidence is accumulated (when V crosses b), as decision is made. The A parameter represents the randomized starting point of evidence for a decision (V can start at any point within A’s boundaries), and t0 represents nondecision time, when participants are first perceiving the stimulus

Cognitive modeling could provide insight into the underlying mechanisms by which these types of competition influence performance. In the current study, we used a speeded choice task, allowing us to use evidence accumulation models to quantify latent processes. These models assume people make decisions by sampling information over time and accumulating evidence until reaching a threshold, which triggers a response (S. D. Brown & Heathcote, 2008; Ratcliff & McKoon, 2008; Usher & McClelland, 2001). In this research, we use the Linear Ballistic Accumulator (LBA) model, shown in Fig. 1 (S. D. Brown & Heathcote, 2008).

The rate of evidence accumulation indexes the speed of information processing (Ballard et al., 2019; Eidels et al., 2010; Palada et al., 2016). Greater differences between the rates for the correct versus incorrect responses indicate greater efficiency of information processing. Within-person variation in this difference can therefore be used as a measure of effort expenditure. The response threshold reflects the decision strategy. Higher thresholds reflect cautious strategies because they allow more evidence to be accumulated before the decision is made, increasing accuracy at the cost of speed. Lower thresholds reflect risky strategies because they produce faster but less accurate decisions. While the competition literature has primarily focused on effort (e.g., Gill & Prowse, 2012; Huang et al., 2017; Kilduff, 2014; Wittchen et al., 2013), far less attention has been paid to how people regulate strategies during competitions.

In this research, we examine how effort and strategy change as a function of competition type. We do this by fitting the LBA model to data from a speeded decision-making task, where correct decisions gain points and incorrect decisions lose points. In this task, participants completed four different competitive schemes: race, tournament, noncompetitive goal pursuit (achieve a certain number of points within a certain deadline), and a non-competitive do-your-best condition (achieve as many points as you can). If consistent with Wittchen et al. (2013), and people respond to a particular competitive scheme by increasing effort, we expect to observe an increase in the difference between the evidence accumulation rates for decisions made in that condition. If people respond by adjusting strategy, we expect to observe a change in the threshold parameter—lower thresholds indicating a sacrifice in accuracy for speed and higher thresholds indicating a sacrifice in speed for accuracy. The model also allows us to assess the moderating role of trait competitiveness on these relationships. For example, to determine whether highly competitive individuals adjust their effort more in the face of competition than less competitive individuals, whether they show more pronounced changes in strategy, both, or neither.

## Method

### Participants

The sample consisted of 100 participants (44 male, 53 female, one preferred not to say, and two nonresponses, mean age of 19.71 years) who were undergraduate psychology students at an Australian university. These individuals participated for course credit. This number of participants meets the sample size requirements for small correlations (in this case, between trait competitiveness and the LBA parameters) with 80% power and an alpha of .05 (Bujang & Baharum, 2016). We expected small correlations since we were correlating model parameters with a personality factor. The Open Science Framework preregistration for this study can be found here (https://osf.io/aqh4v/), and an addendum can be found here (https://osf.io/cg3nj/).

### Experimental task

**Fig. 2:**
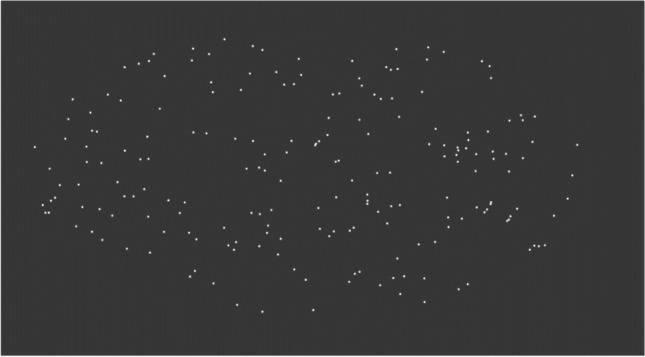
Screenshot of the random dot stimulus

The experiment used the random dot task, a perceptual decision-making task that requires participants to make quick and accurate decisions in response to the visual stimuli (Holmes et al., 2016). Participants were presented with a cloud of rapidly moving dots and indicated whether they were moving to the right or left. Ten percent of the dots moved in the same direction, with the remaining dots moving in random directions. The stimuli consisted of 200 dots that were each four pixels wide in an aperture that is 1,000 pixels wide and 500 pixels high. Each dot moved three pixels per frame and moved along its trajectory (randomly or coherently left/right) for 20 frames before being reset at a random location. The experiment was adapted from code by Rajananda et al. (2018).^1^ A screenshot of the task is shown in Fig. 2.

**Fig. 3:**
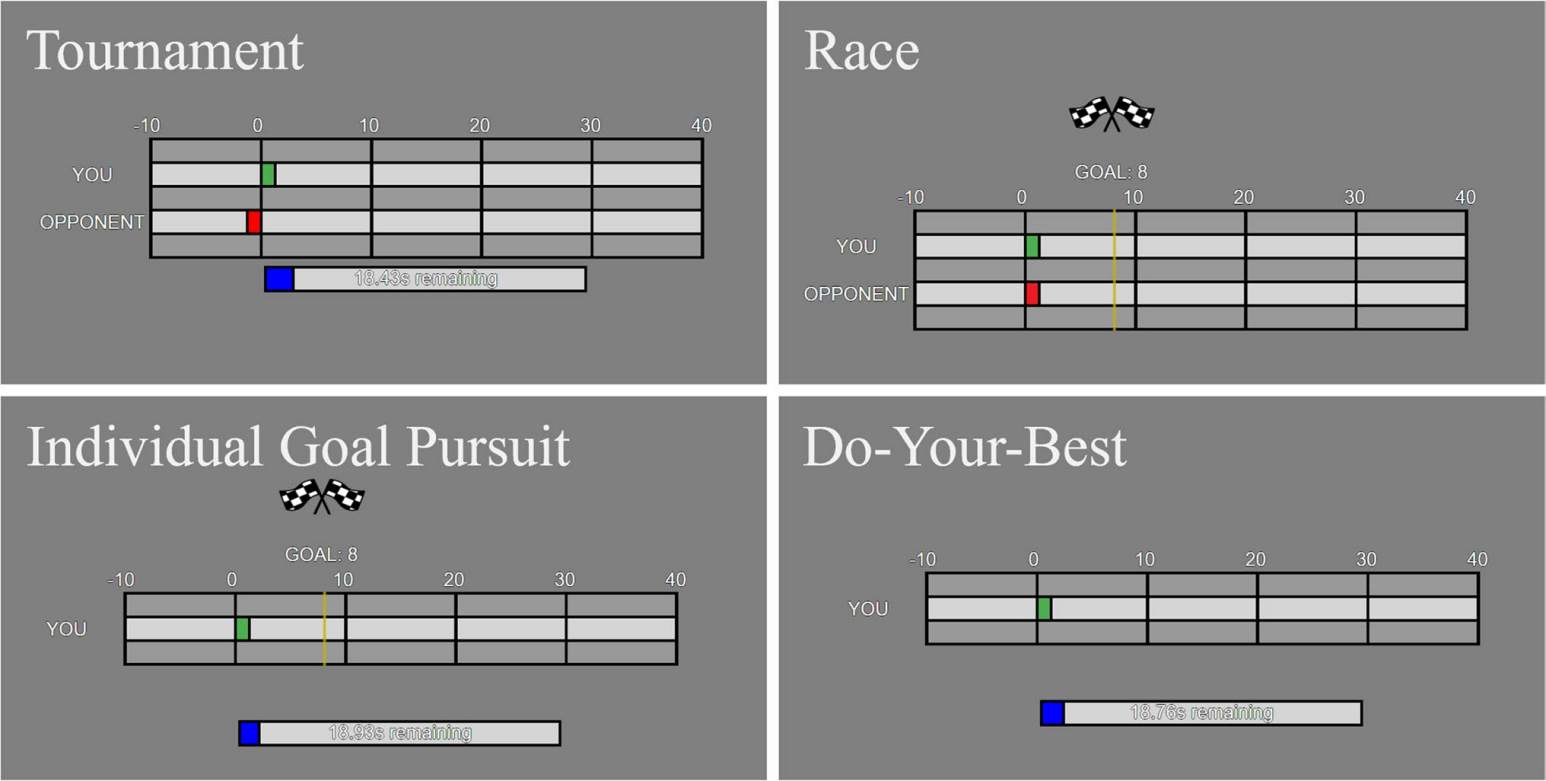
Feedback screens for the tournament, race, individual goal pursuit, and do-your-best conditions (condition labels are not included in the actual experiment). The participant’s score is shown in green (the "YOU" bar), the opponent’s score is shown in red (the "OPPONENT" bar), the elapsed time is shown in blue (the bottom bar), and the goal is shown in yellow (the line across the score display). (Color figure online)

These decisions were made using a keypress input on a keyboard. The participants pressed the “A” key if they believed the coherent dots were moving left and the “L” key if they believed the coherent dots were moving right. Each correct decision earned the participant a point while each incorrect decision lost them a point. After each decision, they were shown a progress screen for one second indicating their score, and, where relevant, their opponent’s score, the goal, and the time remaining (Fig. 3). Once the time limit had run out or the goal had been achieved, the participants were shown the feedback screen and told whether they had been successful or not. They also received a prompt to press the “R” key to begin the next competition.

### Measures and manipulations

#### Trait competitiveness

The Revised Competitiveness Index was used to assess participants’ trait competitiveness (Houston et al., 2002). The index consists of 14 items asking participants the extent to which they agree with a statement on a five-point Likert-type scale (1 = *strongly disagree* and 5 = *strongly agree*). Nine of the items relate to the enjoyment of competition (e.g., “I enjoy competing against an opponent”), while the other five assess conscientiousness (e.g., “I try to avoid arguments”). The scores were averaged together to form a trait competitiveness score.

#### Competition type

Competition type was manipulated across four different levels: tournament, race, individual goal pursuit (IGP), and do-your-best (see Table 1). In the tournament condition, participants competed against an opponent to accumulate the most points at the end of a 20-second time window. In the race condition, participants competed against an opponent to be the first to accumulate 8 points. In the tournament and race conditions, participants were told the opponent’s performance was determined based on data from real participants in a pilot study. The decisions made by the computerized opponent were generated using the LBA, with parameter values determined by fitting the model to pilot data and aggregating across participants. For the IGP condition, participants had the goal of accumulating 8 points in 20 seconds, without an opponent. For the do-your-best condition, participants simply tried to accumulate as many points as they could in 20 seconds (also without an opponent). The difficulty of the task was calibrated such that beating the opponent in the race or tournament was about equally difficult as achieving the goal in the goal pursuit condition (opponents would take approximately 20 seconds to achieve a goal of eight).

**Table 1:** The four competition conditions and their respective features

	Tournament	Race	Individual goal pursuit	Do-your-best
Deadline	✔		✔	✔
Goal		✔	✔	
Opponent	✔	✔		

#### Procedure

Participants completed the task in a laboratory. After reading a brief description of the study, they completed the Revised Competitiveness Index and were asked to provide demographic information. Participants were then instructed on how to complete the task and completed a practice episode of the task. For each condition, participants were told their objective and completed 15 episodes of that competition type. All four competition types were completed in a random order. After completing conditions, they were debriefed about the task and given details on how to find out more about the study.

The sample size was 90,180 total decisions across 6,000 competitive episodes (60 per participant), meeting the recommended sample size for accurate parameter estimation through the LBA (Donkin et al., 2009). Excluded from the analyses were the practice rounds and, in line with our preregistration, decisions that took 250 ms or less or took 5 seconds or more. Response times that are too quick suggest the participant did not actually make a decision, whereas those that were too long are suggestive of attentional lapses. After applying the exclusion criteria, 7.30% of decisions were removed. The code for the experiment and the analyses, the data, and supplementary materials are available publicly on the Open Science Framework (https://osf.io/4xupt).

This project met the ethical requirements for conducting human research and legal requirements in Australia. It was approved by The University of Queensland Human Research Ethics Committee B, approval number 2018002018.

## Results

We first examined the effects of competition type and trait competitiveness on accuracy and response time, and then used the LBA to examine how competition type affected threshold and rate of evidence accumulation. The LBA model was implemented in Stan within a hierarchical Bayesian framework (Annis et al., 2017; Carpenter et al., 2017). Threshold and the difference in rates could vary across competition type, and the sum of the rates, nondecision time, and starting point variability parameters were constrained to be equal across conditions. We used the rate difference between the correct and incorrect accumulators as our measure of effort, as this reflects the efficiency with which the decision maker differentiates signal from noise (with more cognitive resources allowing more rapid evidence accumulation for the correct decision and less for the incorrect decision, (Eidels et al., 2010). Priors were based on Gronau et al. (2020). The Savage–Dickey density ratio (Verdinelli & Wasserman, 1995) was used to compute Bayes factors (BF) for the LBA parameters. Lee and Wagenmakers’ (2013) classifications were used to describe evidence strength. The 95% credible interval (CI) is also reported for each effect to evaluate magnitude. Further details on the analyses can be found in the supplementary materials.

### Effects on accuracy and response time

**Fig. 4:**
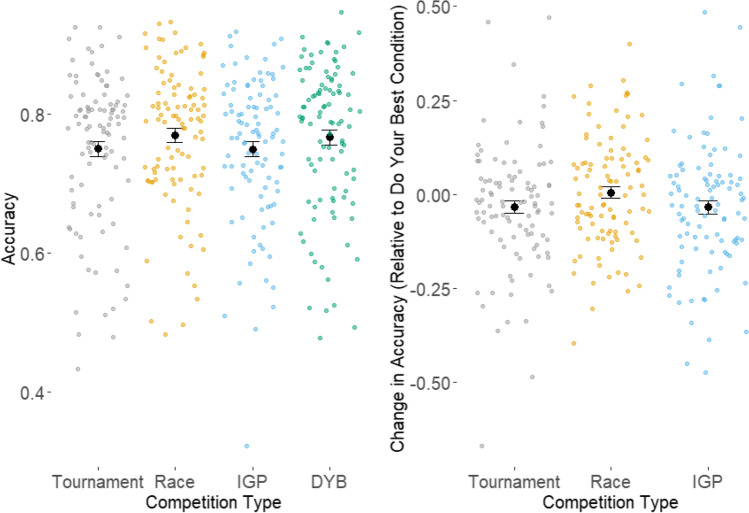
Effects of competition type on accuracy. For the left graph, the black points represent the mean change in accuracy across individual participants by condition. The colored points represent the mean change in accuracy of the individual participants across each condition. For the right graph, the black points represent the mean change in accuracy across individual participants by condition, compared against the do-your-best condition. The colored points represent the mean change in accuracy of the individual participants across each condition, compared against the do-your-best condition. (Color figure online)

Three dummy-coded variables were created to mark the tournament, race, and IGP conditions (1 if the data belonged to that condition, 0 if not). A Bayesian mixed-effects regression was run using R and brms (Bürkner, 2017; R Core Team, 2021) with these variables and trait competitiveness as predictors. The condition variables and the participant identifiers were entered as random effects. The effects of the dummy-coded variables reflect the differences between the do-your-best condition and the condition marked by that variable. The intercept in the model corresponded to the mean in the do-your-best condition. The Bayes factors for the regression models used for the behavioral data were obtained by comparing models with and without each competition type and trait competitiveness via bridge sampling (Gronau et al., 2017). The accuracy model used a logit link function. The mean accuracy in the do-your-best condition was 76.7%, the model intercept estimate was 0.73 (CI [0.19, 1.31]). Figure 4 shows the mean change in accuracy in all conditions, and each condition relative to the do-your-best condition, together with the mean change for each individual in each condition. We found extreme evidence for a difference in accuracy between the tournament and do-your-best conditions, with accuracy being lower in the tournament condition (BF > 1,000, CI [−0.15, −0.07], *M* = 75.1%). We also found strong evidence of no difference in accuracy between the race and do-your-best conditions (BF = 0.03, CI [−0.10, −0.01], *M* = 77.0%). We found moderate evidence a difference in accuracy between the IGP and do-your-best conditions, with accuracy being lower in the IGP condition (BF = 3.34, CI [−0.13, −0.04], *M =* 75.0%). Additionally, we found strong evidence of no association between trait competitiveness and accuracy (BF = 0.05, CI [−0.01. 0.34], *M* = 0.17). For a visual representation of the within-person patterns of accuracy and response time across conditions, please see the supplementary materials.

**Fig. 5:**
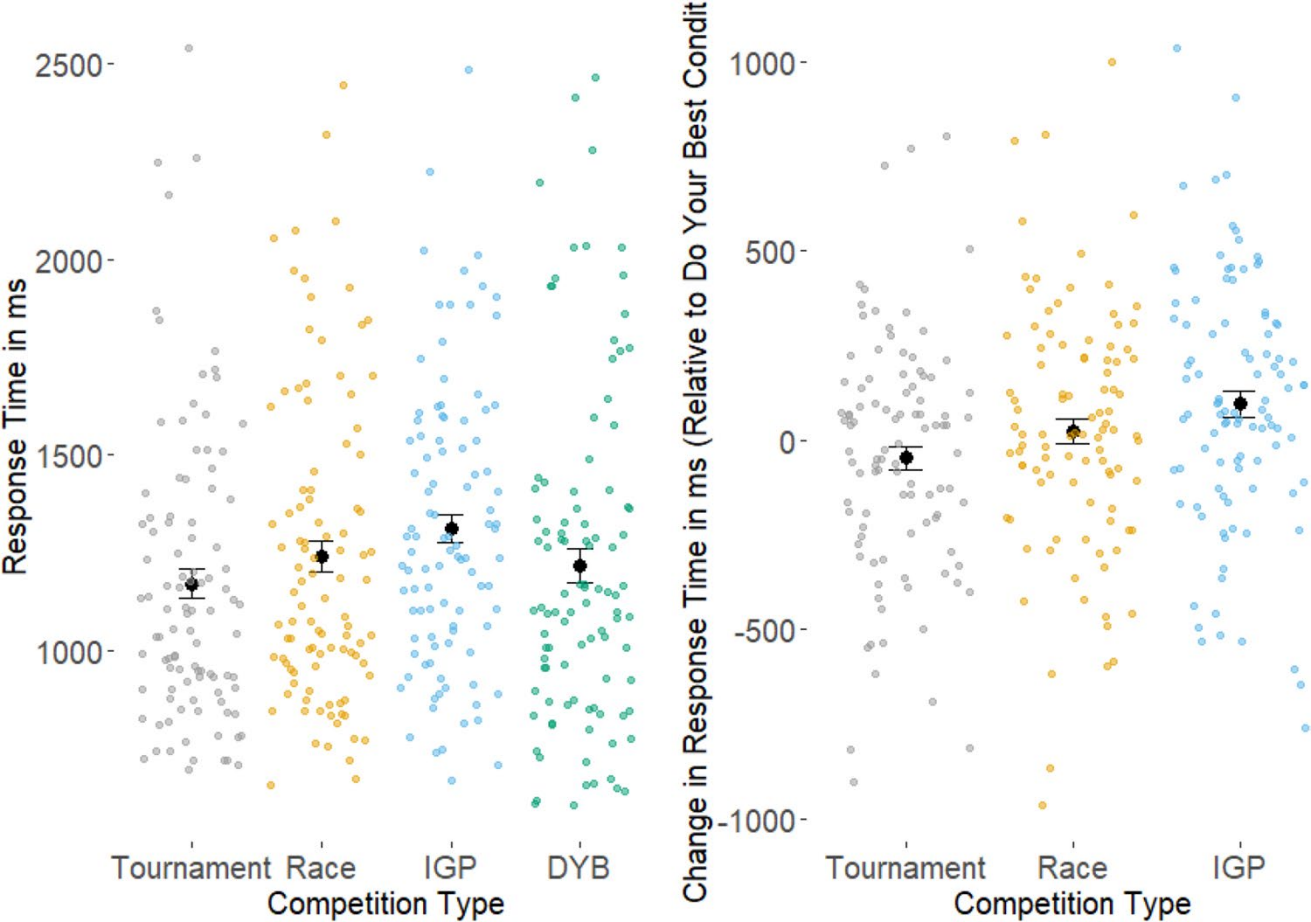
Effects of competition type on response time. For the left graph, the black points represent the mean change in response time across individual participants by condition. The colored points represent the mean change in response time of the individual participants across each condition. For the right graph, the black points represent the mean change in response time across individual participants by condition, compared against the do-your-best condition. The colored points represent the mean change in response time of the individual participants across each condition, compared against the do-your-best condition. (Color figure online)

The model for the response time data was run with a log link function. Figure 5 shows the mean change in response time in all conditions, and each condition relative to the do-your-best condition, together with the mean change for each individual in each condition. The mean response time in the do-your-best condition was 1,217.1 ms, the model intercept estimate was 7.12 (CI [6.83, 7.41]). We found extreme evidence for no difference in response time between the tournament condition and the do-your-best condition, (BF = 0.0004, CI [−0.02, 0.002], *M* = 1169.9 ms). We also found extreme evidence of a difference in response time between the race and do-your-best conditions, with longer response times in the race condition (BF > 10,000, CI [0.06, 0.08], *M* = 1242.0 ms). We found extreme evidence of a difference in response time between the IGP and do-your-best conditions, with longer response times in the IGP condition (BF > 10,000, CI [0.10, 0.12], *M* = 1312.6 ms). Finally, we found extreme evidence for no association between trait competitiveness and response time (BF = 0.004, CI [−0.16, 0.02], *M* = −0.07).

### Posterior predictive fits

**Fig. 6:**
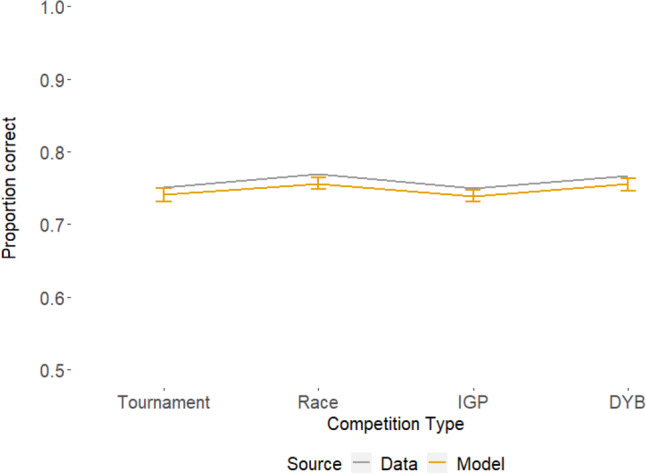
Mean proportions of correct responses for the real data (the line without error bars) and the simulated model data (the line with error bars). The error bars for the predicted data represent the 95% credible interval

**Fig. 7:**
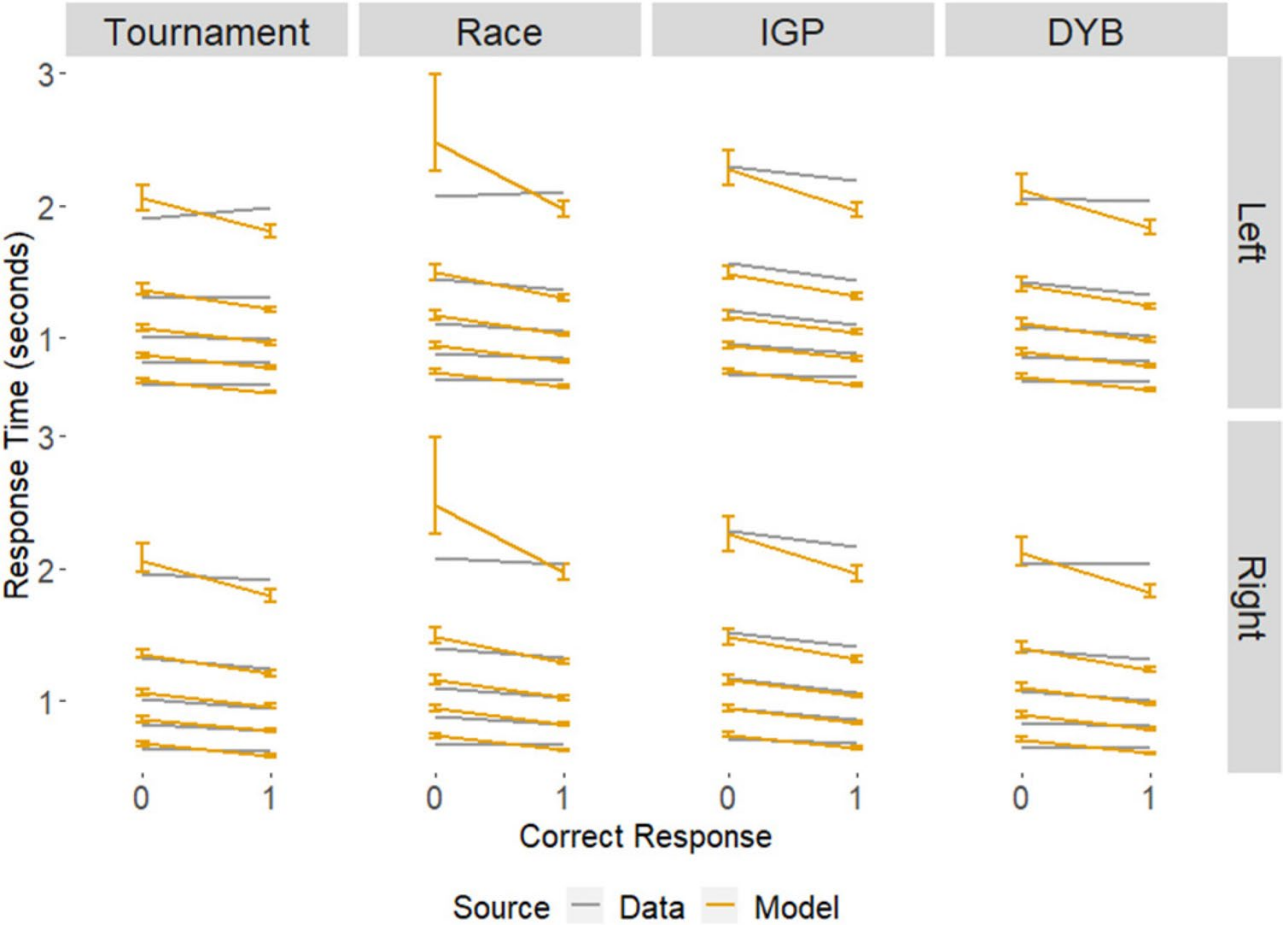
The mean 10^th^, 30^th^, 50^th^, 70^th^, and 90^th^ quantiles for the response time distributions that were observed (the lines without error bars) and predicted by the model (the lines with error bars). The directions (left or right) indicate the response made by the participant. Correct response is shown with 0 being the incorrect response and 1 being the correct response. The error bars for the predicted data represent the 95% credible interval

To check the posterior predictive fit of the model, we visually compared the observed data to predicted data from the model using the estimated parameters. Figure 6 shows the proportion of correct responses for the observed data and model data as a function of competition type. Figure 7 shows the same for response times. To calculate the observed values for both figures, the relevant values were averaged for each participant, and then averaged across participants. The predicted values were calculated through the same procedure for each sample, allowing a full posterior distribution to be obtained for each value. The model provides a relatively close fit to the accuracy data, allowing enough complexity to capture the patterns of the data while still maintaining simplicity. Overall, the model also provided a good account of the response time data, particularly between the 10th and 50th quantiles, though there is some misfit at the 90th quantile.

### Effects on thresholds

**Fig. 8:**
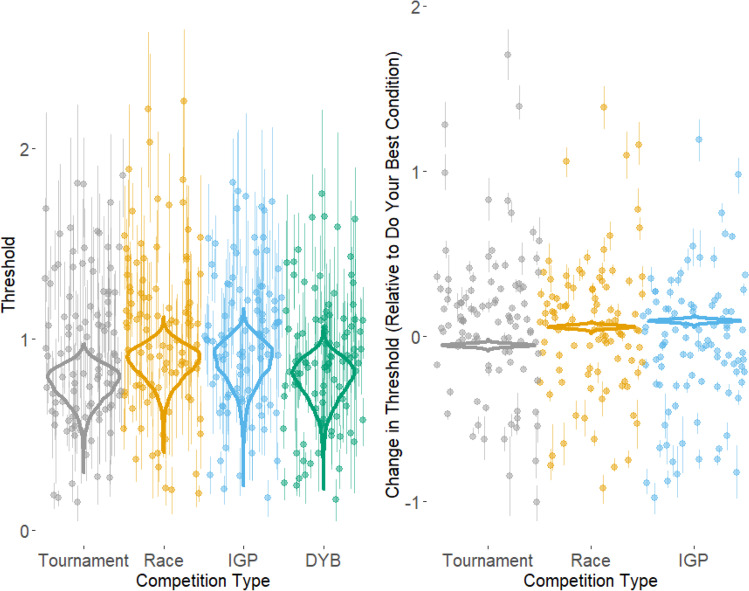
Effects of competition type on threshold. The left graph shows the overall position of the four conditions, while the right graph shows the conditions’ positions relative to the do your best condition. The violin plots represent the posterior distribution of the mean condition parameters across individual participants. The points represent the mean of the posterior distribution of the individual participants

Figure 8 shows the effects of competition type on the threshold parameter. The CI for mean threshold in the do-your-best condition (i.e., the intercept of the model) was [0.92, 0.98], with a mean of 0.95. We found extreme evidence of a difference in threshold between the tournament and do-your-best condition, with lower thresholds in the tournament condition (BF = 656.24, CI [−0.07, −0.04], *M* = 0.89). We also found extreme evidence of a difference in threshold between the race and do-your-best conditions, with higher thresholds in the race condition (BF = 609.98, CI [0.03, 0.08], *M* = 1.00) as well as between the IGP condition and the do-your-best condition, with higher thresholds in the IGP condition (BF = 501.13, CI [0.07, 0.11], *M* = 1.04). A posterior representative analysis was run to assess the individual-level effects, which do not appear to contradict the overall effects found here (see supplementary materials for more details).

### Effects on drift rates

**Fig. 9:**
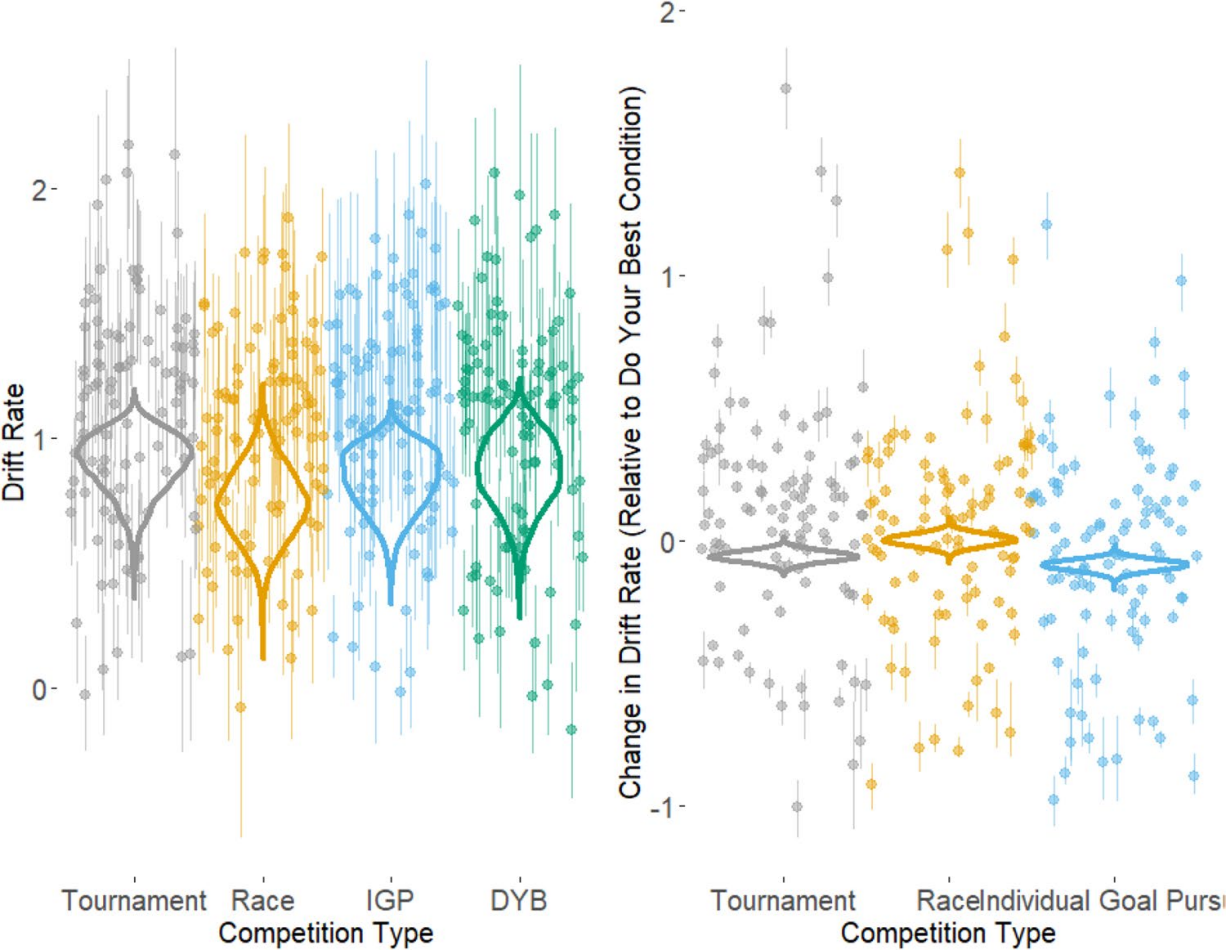
Effects of competition type on drift rate. The left graph shows the overall position of the four conditions, while the right graph shows the conditions’ positions relative to the do your best condition. The violin plots represent the posterior distribution of the mean condition parameters across individual participants. The points represent the mean of the posterior distribution of the individual participants

The effects of competition type on drift rates were examined by assessing how the rate difference between the correct and incorrect response accumulators’ changes across conditions. Figure 9 shows these effects. The CI for mean drift rate in the do-your-best condition was [1.07, 1.13], with a mean of 1.10. We found nondiagnostic evidence for no difference between the tournament condition and the do-your-best condition was found (BF = 0.65, CI [−0.10, −0.02], *M* = 1.04) and extreme evidence of no difference between the race and do-your-best conditions (BF = 0.007, CI [−0.04, 0.05], *M* = 1.10). We found strong evidence for a difference between the IGP and do-your-best conditions, with the IGP condition having a smaller difference in rates (BF = 621.71, CI [−0.13, −0.05], *M* = 1.01).

The effects of threshold and rates were also assessed jointly using the Savage–Dickey method on a joint posterior distribution of the difference in means between each condition and the do-your-best condition (this analysis was not preregistered). The Bayes factors for no effects, effects of only rates or threshold, or both effects are shown in Table 2. The results for the race and IGP conditions were consistent with the individual analyses, suggesting only threshold is affected in the former, but both threshold and rates are affected in the latter. The results for the tournament suggested both threshold and rates were affected (the individual analyses revealed a threshold effect but the rate effect was non-diagnostic).

**Table 2:** Joint Bayes factors for the effects of competition type on change in threshold and drift rate (relative to the do-your-best condition)

Condition	Hypothesis 1	Hypothesis 2	Bayes Factor (H1/H2)
Tournament	No effects	At least one effect	<0.0001
	Effect on threshold only	No effects, or an effect on both	5.73
	Effect on drift rate only	No effects, or an effect on both	<0.0001
	Effect on both	Only one effect or no effects	31.94
Race	No effect	At least one effect	<0.0001
	Effect on threshold only	No effects, or an effect on both	643.08
	Effect on drift rate only	No effects, or an effect on both	<0.0001
	Effect on both	Only one effect or no effects	46.37
Individual goal pursuit	No effect	At least one effect	<0.0001
	Effect on threshold only	No effects, or an effect on both	0.0001
	Effect on drift rate only	No effects, or an effect on both	<0.0001
	Effect on both	Only one effect or no effects	44.73

The no-effect comparison measured whether the density where both quantities equal zero increased (H1) or decreased (H2). The comparison for an effect on threshold only examined whether the density corresponding to a change in threshold and no change in drift rate increased (H1) or decreased (H2). The comparison for an effect on drift rate only examined whether the density corresponding to a change in drift rate and no change in threshold increased (H1) or decreased (H2). The comparison for an effect on both threshold and drift rate looked at the change in density corresponding to a chance in both quantities increased (H1) or decreased (H2). Bayes Factors in favor of H1 are above 1, while Bayes factors in favor of H2 are below 1

### Effects of trait competitiveness

We investigated the effects of trait competitiveness by including it as a participant-level covariate in the LBA model, following the approach developed by Boehm et al. (2018). The model estimated the association between trait competitiveness with threshold and rate difference separately for each competition type. As can be seen in Table 3, moderate to strong evidence was found for no effect on both threshold and rate difference in almost all conditions. The evidence of an effect on drift rates in the race condition was non-diagnostic.

**Table 3:** Bayes factors and 95% credible intervals for the relationships of trait competitiveness with thresholds and drift rates in the four competition conditions

Parameter	Condition	BF	Lower CI	Upper CI
Threshold	Tournament	0.12	−0.12	0.07
	Race	0.07	−0.11	0.09
	Individual goal pursuit	0.08	−0.12	0.12
	Do-your-best	0.08	−0.11	0.12
Drift rate difference	Tournament	0.07	−0.06	0.14
	Race	1.30	0.03	0.22
	Individual goal pursuit	0.09	−0.03	0.15
	Do-your-best	0.19	−0.02	0.18

## Discussion

This research sought to integrate work from disparate literatures—competition and decision-making—to understand the effects of different competition types and trait competitiveness on the information processing underlying rapid choice during competitions. To do so, we used the LBA model to quantify effort and strategies used when faced with competitions of different structure, as well as their relationships with trait competitiveness.

When faced with an opponent, participants responded less cautiously (relative to the do-your-best condition) in tournaments and more cautiously in races. This demonstrates different types of competitions impacting behavior in different ways. One possible explanation for the difference in responding between these conditions concerns differences in the nature of the uncertainty created by the opponent. In the tournament condition, where participants had to score more points than the opponent within a fixed deadline, the opponent creates uncertainty in the points needed to win. In the race condition, where participants had to reach a fixed-point target before the opponent, the opponent creates uncertainty in the time within which the target must be reached. It is possible the fixed deadline in the tournament context makes the time pressure more salient, leading to less cautious responding. Previous work has shown concrete deadlines decrease response caution (Dambacher & Hübner, 2015). By contrast, the uncertainty in the deadline created by the race context may have made the time pressure less salient, encouraging participants to respond more cautiously. The tendency to set lower thresholds in the tournament condition is consistent with findings that competition indirectly increases risk-taking (Hangen et al., 2016), as lower thresholds are associated with less caution. This tendency, however, did not extend to the race condition, suggesting the relationship between competition and risk-taking may depend on the competition structure.

While we were able to identify differential strategies between the do-your-best and competition conditions, no effects on effort were found between these conditions. Previous studies have found effects of competition on behavioral indicators of effort, such as time spent on task and resource investment (Huang et al., 2017; Zizzo, 2002). It is possible these behavioral indicators reflect the strategies participants use, rather than effort. For example, higher time or resources spent may be more indicative of a cautious strategy than increased effort. Our findings suggest people are more likely to adjust their strategy in response to competition, than to adjust effort. These findings are consistent with previous studies that used cognitive modeling to examine how people adapt to time pressure (Palada et al., 2018).

One reason why effort may have been sensitive to competition has to do with the task used. Changes in the amount of effort expended in the random dot discrimination task may not necessarily lead to large changes in performance. The discrimination decision relies on automatic perceptual processes that are not necessarily under conscious control. If they were, we would expect a stronger relationship between competition type and effort in tasks where effort expenditure improves performance. For example, sales contests between employees (Casas-Arce and Martinez-Jerez, 2009) could require competitors to put greater conscious effort into making a sale. This gives pause in extending the findings on effort to other competitive tasks, especially those with a higher reliance on conscious control.

Compared with the do-your-best condition, participants were more cautious and applied less effort during IGP. This is inconsistent with findings that participants select risker gambles when given a goal, compared with being told to do their best (Larrick et al., 2009). These effects may be explained by the assigned goal being easier than the participants’ self-set goals in the do-your-best condition. If the goal required participants to accumulate fewer points than they would have otherwise aimed for with no explicit goal, the goal may have undermined motivation to perform, resulting in decreased effort (Vancouver et al., 2020). In this case, the participant would also experience less time pressure in the IGP condition, as they could afford to accumulate points more slowly. This would allow participants to respond more cautiously and accumulate more evidence before responding (Dambacher & Hübner, 2015).

The investigation into the impact of trait competitiveness provided no meaningful evidence of effects on strategy and effort, suggesting an individual’s competitiveness did not change how they made decisions across competition types. Yet trait competitiveness has been shown to moderate the relationship between competition climates and performance in actual workplaces (Fletcher et al., 2008). It is possible the effects of trait competitiveness only emerge in settings where decisions have more meaningful consequences.

The notion of an optimal threshold that maximizes performance through balancing speed and accuracy is also important. Addressing this is challenging, as there are multiple ways to quantify performance. Looking at win rate, the optimal threshold to win changes in competitive conditions with the opponent’s performance. Winning is also irrelevant in the do-your-best condition. Another option is points scored, though this does not fit the race and IGP conditions with their upper point limits. These challenges make it difficult to analyze threshold optimality (though see supplementary materials for a preliminary analysis of threshold optimality).

One lingering question not addressed by our study is how effort and strategy change over time as the competition unfolds and in response to looming deadlines and changes in relative progress. We now know people may respond to different types of competition in different ways, but these responses likely change over time. For example, a day trader who tries to maximize profit each day may make quicker decisions to trade as time runs out, or as their profit gets closer to an opponent’s. Examining the dynamics of how effort and strategy respond to competition could allow for a more nuanced understanding of competitions over time. This could be done using methodology that accounts for switching strategies using spike-and-slab priors (Lee, 2019; Lee & Gluck, 2021). Other work could look at applying evidence accumulation modeling to other work in decision-making. Hintze et al. (2015) found that competition strength influenced strategy, where extreme competitions (where points are taken from the opponent) had quicker decisions. It is possible this could be another impact of competition structure on decision-making, and the current competition types could be changed to include a point-stealing component. Phillips et al. (2014) found that expectations about opponent decision speeds influences search time, which could also have some impact on strategy through manipulation of opponent speed.

There is also the question of whether people use collapsing bounds when making these decisions. Previous research suggests this could be the case, particularly when decision difficulty is variable and people need to be strategic about which stimuli they spend time on (Malhotra et al., 2017; Malhotra et al., 2018). In these cases, people may use a collapsing threshold to ensure they do not spend too much time on stimuli that are more challenging to discriminate. The difficulty was fixed in the current research, but a potential next step for future work would be to examine the potential for collapsing bounds in a paradigm where difficulty is higher or less predictable.

## Supplementary information

ESM 1 (DOCX 457 kb)

## Data Availability

All data for this experiment can be found online (https://osf.io/4xupt/).

## Footnotes

1 While we have used a modified version of Rajananda et al.’s (2018) random dot stimulus task, many previous studies are based on Newsome and Pare’s (1988) random dot stimulus task. The major difference between the tasks is a less even distribution of dots in the current task, which could lead to greater variability in performance.

## Code availability

The experimental code for this experiment can be found online (https://osf.io/4xupt).
