# Supplementary material for: Using cognitive modeling to examine the effects of competition on strategy and effort in races and tournaments
Source: Psychon Bull Rev. 2022 Nov 16;30(3):1158–69. doi: 10.3758/s13423-022-02213-x (PMC10264533; doi:10.3758/s13423-022-02213-x)

**Pilot Study**

In order to develop the stimuli for the experiment, we ran four pilot studies to determine an appropriate difficulty for the random dot discrimination and the computerized opponents. The first pilot test included three trials of ten different proportions of dots moving coherently in the Random Dot Motion Task. The coherences ranged from .1 to 1 in incremental steps of .1. Participants had 20 seconds to achieve a goal of 10 points and were not competing with an opponent. After having 22 undergraduate participants complete the pilot, we observed the data to find a coherence that led to approximately 70% accuracy and goal achievement 50% of the time. Accuracy and response times are shown below in Figure 1.

**Figure 1**

*Response time and accuracy data from the first pilot study.*


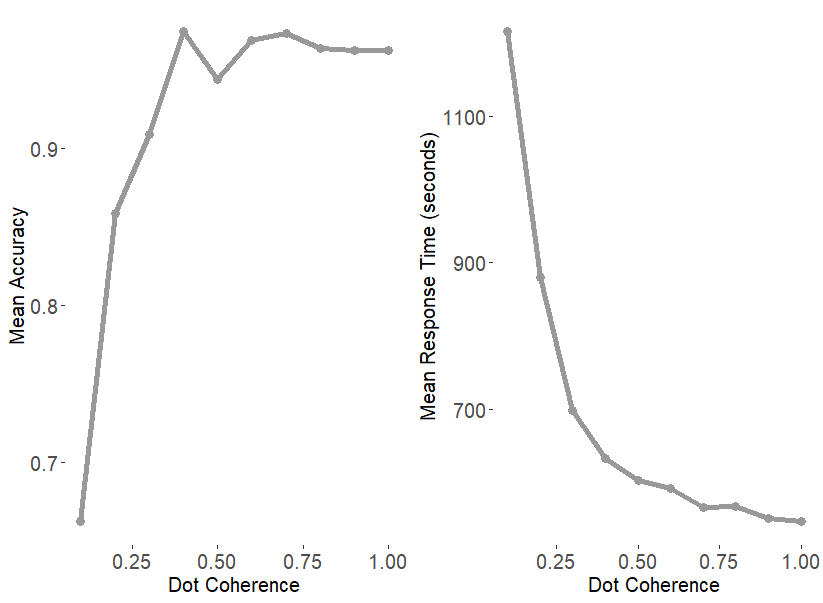


We concluded that the desired difficulty occurred somewhere below .2 and proceeded to pilot test the coherences from .04 to .26 in steps of .01. Each coherence had two episodes and maintained the time limit and goal from the first pilot. This second pilot had a sample of 23 undergraduate participants, and the accuracy and response times are shown below in Figure 2.

**Figure 2**

*Response time and accuracy data from the second pilot study.*
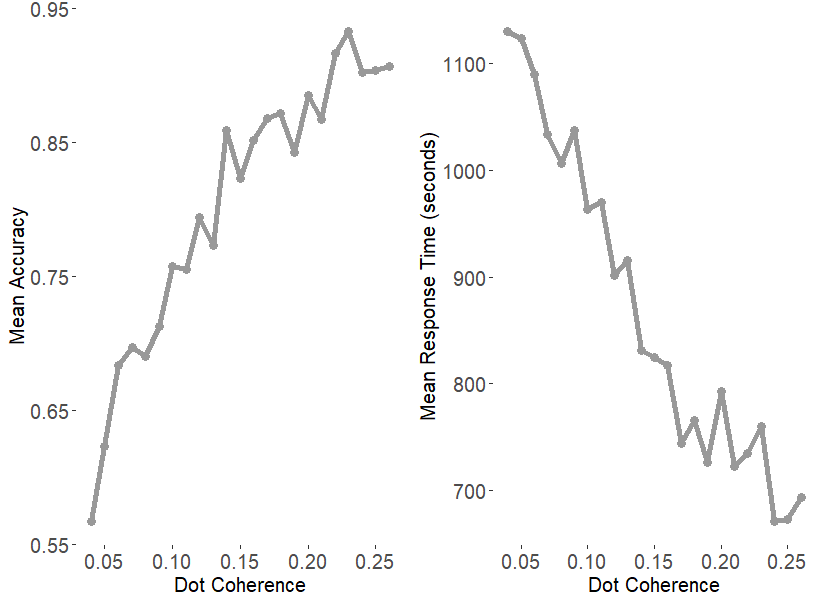


The .075 coherence trials appeared to have the required difficulty and was used for the next pilot. We had 12 undergraduate participants complete 30 trials at .075 coherence. The time limit remained at 20 seconds, though the goal was decreased to eight points, as approximately 54.7% of trials with coherences of .07 and .08 ended with at least eight points, roughly meeting our desired 50% goal achievement criteria. The purpose of this third pilot was determine an appropriate difficulty for the computer opponent, based on a model of how the average participant would perform on the task. Upon analyzing the data, however, it appeared this coherence was more difficult than anticipated, with only 19.4% of trials ending in the participant achieving the set goal. This prompted us to lower the difficulty by raising the coherence to .1, and we ran another pilot study. We had ten undergraduate participants complete another 30 trials with this coherence with the same time limit and goal. Based on the data, we concluded that this was an appropriate difficulty for the task. Data from the third and fourth pilot studies are shown in Table 1, below. We then fitted the data from the final pilot to the LBA. The LBA parameters used for stimulating the opponent in the actual experiment are outlined below in Table 2. Each parameter’s mean and standard deviation had its own mean and standard deviation, which were used to randomly sample parameter values for each competitive episode.

**Table 1**

*Average response time, accuracy, and goal achievement rate for the third and fourth pilots.*

|  | Pilot 3 | Pilot 4 |
| --- | --- | --- |
| % of Goals Achieved | 19.4% | 46.3% |
| Average Accuracy | 60.2% | 73.9% |
| Average Response Time | 1.206 | 1.419s |

**Table 2**

*LBA parameter values for the computer opponent*

| Parameter | Mean | SD | Truncated |
| --- | --- | --- | --- |
| B Mean | 0.08 | 0.05 | Yes |
| B SD | 1.02 | 0.26 | Yes |
| A Mean | 1.06 | 0.38 | Yes |
| A SD | 1.86 | 0.43 | Yes |
| V True Mean | 1.72 | 0.22 | No |
| V True SD | 0.66 | 0.23 | Yes |
| V False Mean | 0.78 | 0.19 | No |
| V False SD | 0.57 | 0.18 | Yes |
| Tau Mean | 0.13 | 0.02 | Yes |
| Tau SD | 0.06 | 0.05 | Yes |
| S | 1 | - | - |

**Statistical Analyses**

We examined the effects of competition types and trait competitiveness on response time and accuracy by conducting Bayesian mixed effects models using Bürkner’s (2017) brms package for R. The effects of the different competition types were modeled using three dummy-coded variables. Data from the tournament condition were coded as 1 for the tournament variable, data from the individual goal pursuit condition were coded as 1 for the individual goal pursuit variable, and data from the race condition were coded as 1 for the race variable. If data did not belong to the respective condition’s variable, they were coded as 0. Cases where all three of the above variables had values of 0 represented the do your best condition. These models included the participant as a random effect. One model examined the effects on accuracy (1 = correct, 0 = incorrect) using a logit link function, and the other model examined the effects on response time in milliseconds using a log link function.

The brms R package estimates parameters using the MCMC routine implemented by the programming language Stan (Carpenter et al., 2017). Weakly informative priors were set for these analyses. For all the accuracy model priors, a Student’s *t*-distribution was used where df = 3, location = 0, and scale = 10. The response time model used the same priors for the fixed effects, whereas the intercept and standard deviation parameters used a *t*-distribution where df = 3, location = 0, and scale = 500. Random effects had a lower bound of 0 and the fixed effects were not truncated. Each model ran four chains with 4000 samples each, where the first 2000 samples for each chain being discarded as warmup. Using the remaining samples, each analysis was based on 8000 samples. Visual inspection of the chains showed excellent mixing, with all R-hat statistics being below 1.06. The results from the accuracy analysis are presented in Table 3, below, while the outputted results from the response time analysis are presented in Table 4, below.

**Table 3**

*Results of the Bayesian Mixed Modelling Analysis of Accuracy.*

|  | Estimate | SE | Lower CI | Upper CI | Bulk ESS | Tail ESS | Rhat | BF |
| --- | --- | --- | --- | --- | --- | --- | --- | --- |
| Intercept | 0.73 | 0.28 | 0.11 | 1.31 | 644 | 939 | 1.01 | - |
| Tournament | -0.11 | 0.02 | -0.15 | -0.07 | 6681 | 6319 | 1.00 | 1082 |
| Race | -0.05 | 0.02 | -0.10 | -0.01 | 7118 | 5779 | 1.00 | 0.03 |
| IGP | -0.09 | 0.02 | -0.13 | -0.04 | 6458 | 6065 | 1.00 | 3.34 |
| Trait Competitiveness | 0.17 | 0.09 | -0.01 | 0.34 | 619 | 833 | 1.01 | 0.05 |
| Intercept SD | 0.50 | 0.04 | 0.43 | 0.58 | 707 | 1365 | 1.01 | - |

SE represents the standard error. Lower CI and Upper CI represent the lower and upper bounds on the 95% credible interval. Bulk and Tail ESS represent the effective sample size, which measures the sampling efficiency in the bulk and tails of the distribution. Rhat represents the R-hat convergence diagnostic, which is a comparison of the between- and within-chain estimate for the model parameters. BF represents the Bayes factor.

**Table 4**

*Results of the Bayesian Mixed Modelling Analysis of Response Time (ms)*

|  | Estimate | SE | Lower CI | Upper CI | Bulk ESS | Tail ESS | Rhat | BF |
| --- | --- | --- | --- | --- | --- | --- | --- | --- |
| Intercept | 7.12 | 0.15 | 6.83 | 7.41 | 71 | 241 | 1.06 | - |
| Tournament | -0.007 | 0.004 | -0.02 | 0.002 | 1625 | 3649 | 1.00 | 0.0004 |
| Race | 0.07 | 0.005 | 0.06 | 0.08 | 1593 | 2718 | 1.00 | 8e+38 |
| IGP | 0.11 | 0.004 | 0.10 | 0.12 | 1592 | 2578 | 1.00 | 3.59e+129 |
| Trait Competitiveness | -0.07 | 0.05 | -0.16 | 0.02 | 68 | 174 | 1.06 | 0.004 |
| Intercept SD | 0.25 | 0.02 | 0.22 | 0.29 | 163 | 331 | 1.03 | - |

SE represents the standard error. Lower CI and Upper CI represent the lower and upper bounds on the 95% credible interval. Bulk and Tail ESS represent the effective sample size, which measures the sampling efficiency in the bulk and tails of the distribution. Rhat represents the R-hat convergence diagnostic, which is a comparison of the between- and within-chain estimate for the model parameters. BF represents the Bayes factor.

Figure 3 shows the relative change in accuracy between the do your best condition and the other conditions. The lines show each participant’s change in accuracy across the three conditions, color-coded to show the overall pattern of their data. Fifty-one participants had the highest change in accuracy in the race condition (gray), twenty-two participants had the lowest change in accuracy in the race condition (yellow), fourteen participants had the highest change in accuracy in the individual goal pursuit conditions (blue), and thirteen participants had the highest change in accuracy in the tournament condition.

**Figure 3**

*Within-person data for accuracy linked across condition, relative to the do your best condition.*


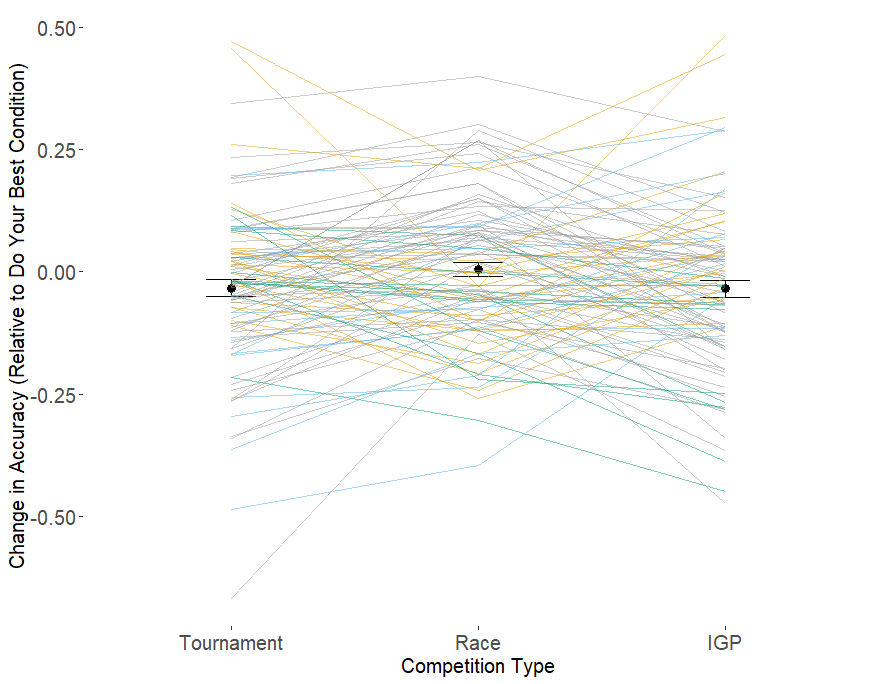


Figure 4 shows the relative change in response time between the do your best condition and the other conditions. The lines show each participant’s change in response time across the three conditions, color-coded to show the overall pattern of their data. Thirty-three participants had the highest change in response time in the race condition (gray), twenty-six participants had the lowest change in response time in the race condition (yellow), thirty participants had the highest change in response time in the individual goal pursuit conditions (blue), and eleven participants had the highest change in response time in the tournament condition.

**Figure 4**

*Within-person data for response time linked across condition, relative to the do your best condition.*


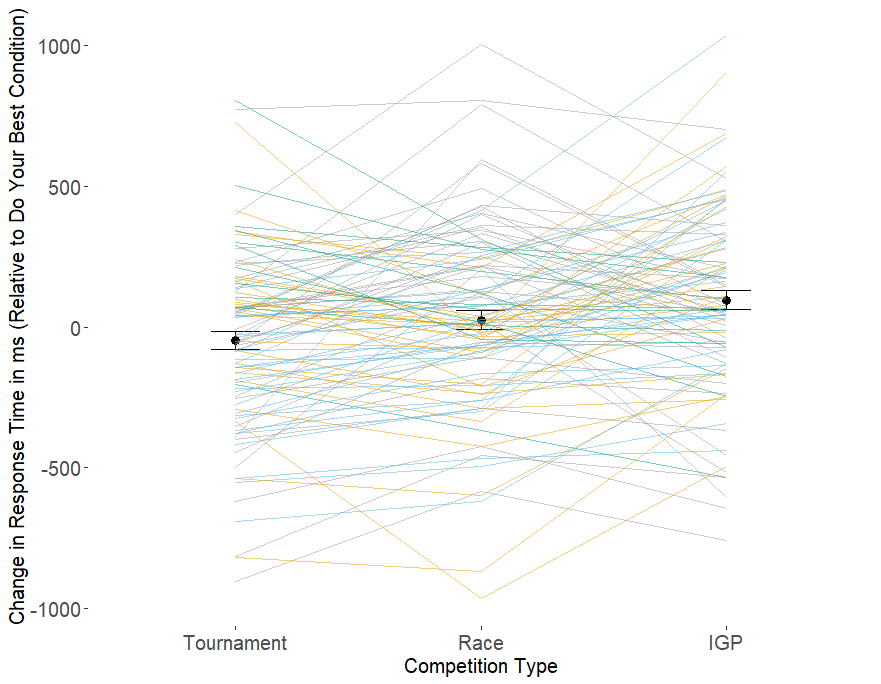


**Computational Modelling**

The Linear Ballistic Accumulator (LBA) model holds the assumption that evidence for either response alternative accumulates in separate accumulators independently of the other. In this experiment, there were two possible responses, left or right, giving two evidence accumulators. The starting evidence for either alternative response for each decision trial is taken from a uniform distribution [0, *A*]. From this starting point, the evidence accumulates linearly. Each accumulator has a rate of evidence accumulation (the drift rate), which is drawn from a normal distribution with mean *v* and standard deviation *sd*. Evidence is accumulated until enough evidence for one response breaches the threshold (*b*) for a decision to be made, at which point, the response is made. In line with common practice (e.g., Brown and Heathcote, 2008), we express threshold as the difference between the raw threshold and the maximum starting evidence (*B*, where *B* = *b* – *A*). This allows for threshold to be measured purely, without contamination by individual differences in starting evidence. In addition, the LBA also includes a parameter for non-decision time (*t_0_*), which captures the portion of response time that is attributed to other processes besides the decision-making process. These include encoding the stimulus and executing the response manually.

In this experiment, a version of the LBA was used where the difference in the mean rates of evidence accumulation (the rate for the correct accumulator minus the rate for the incorrect accumulator) varied as a function of competition type. It also assumed that threshold (*B*) varied as a function of competition type. The starting point variability (*A*), non-decision time (*t_0_*), and the sum of the mean rates of evidence accumulation for correct and incorrect responses were constrained across conditions to be equal. The standard deviation of the drift rate (*sv*) was fixed to one for all conditions and accumulators.

The parameters were estimated using a hierarchical Bayesian framework that assumed that parameters varied across individuals and were drawn from shared population distributions (see Table 5). These population distributions for each parameter have two hyperparameters, being location (μ) and scale (σ). The hyperparameters were chosen to be weakly informative. The parameters at the participant level were modeled using either normal or truncated normal distributions. Both the *A* and *B* parameters were set with a lower bound of 0 and no upper bound. The *t_0_* parameter was constrained between 0.1 and 1, while the *v* parameter was not truncated at all. We analysed the impact of trait competitiveness on threshold and drift rate by allowing the low-level priors to vary as function of the standardised trait competitiveness variable, in with the method from Boehm, Steingroever, and Wagenmakers (2018). The priors used were based on methods used by Gronau, Heathcote, and Matzke (2019).

**Table 5**

*Priors for the Population Distributions.*

| Population Distribution | Model Parameter | Distribution Family | Mean | SD | Lower | Upper |
| --- | --- | --- | --- | --- | --- | --- |
| Location | *A* | Truncated Normal | 1 | 1 | 0 | None |
|  | *B* | Truncated Normal | 0.4 | 0.4 | 0 | None |
|  | *v Sum* | Normal | 4 | 4 | None | None |
|  | *v Difference* | Normal | 2 | 2 | None | None |
|  | *t_0_* | Truncated Normal | 0.3 | 0.3 | 0.1 | 1 |
|  | Trait Competitiveness on *B* | Normal | 0 | 1 | None | None |
|  | Trait competitiveness on *v Difference* | Normal | 0 | 1 | None | None |
| Scale | *A* | Truncated Normal | 1 | 1 | 0 | None |
|  | *B* | Truncated Normal | 0.4 | 0.4 | 0 | None |
|  | *v Sum* | Truncated Normal | 4 | 4 | 0 | None |
|  | *v Difference* | Truncated Normal | 2 | 2 | 0 | None |
|  | *t_0_* | Truncated Normal | 0.3 | 0.3 | 0 | None |
|  | Trait Competitiveness on *B* | Truncated Normal | 0 | 1 | 0 | None |
|  | Trait competitiveness on *v Difference* | Truncated Normal | 0 | 1 | 0 | None |

The posterior distributions were estimated using the Hamiltonian Monte Carlo algorithm implemented by Stan (Annis, Miller, & Palmeri, 2016; Carpenter et al., 2017). Four chains were run, each with 4000 iterations. 2000 of these iterations were run for sample burn in, and were discarded, leaving 2000 iterations. Visual inspection of the chains showed excellent mixing and stationarity.

Figure 5 shows the proportion of correct responses for both the observed and predicted data as a function of competition type. Figure 6 shows the same, except for response times. To calculate the observed values for both figures, the relevant values were averaged for each participant, and these were then averaged across participants. The predicted values were calculated through the same procedure for each sample, allowing a full posterior distribution to be obtained for each value. The model provides a relatively close fit to the accuracy data, allowing enough complexity to capture the patterns of the data while still maintaining simplicity. Overall, the model also provided a good account of the response time data, particularly between the 10th and 50th quantiles. There is some misfit at the 90th quantile, though this is often found in the response time modeling as there are fewer observations at the tail end of response time distributions (Palada et al., 2016).

**Figure 5**

*Mean prorportions of correct responses for the real data and the simulated model data. The error bars for the predicted data represent the 95% credible interval.*


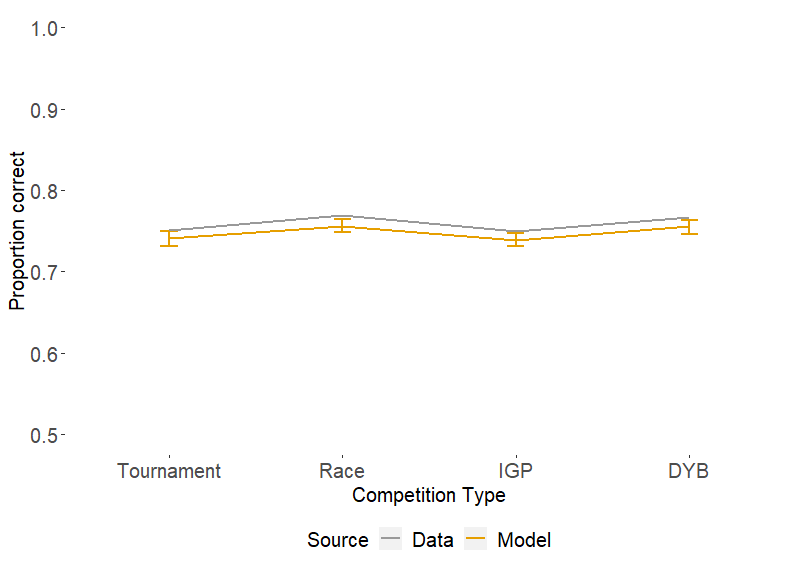


**Figure 6**

*The mean 10^th^, 30^th^, 50^th^, 70^th^, and 90^th^ quantiles for the response time distributions that were observed and predicted by the model. The directions (left or right) indicate the response made by the participant. Correct response is shown with 0 being the incorrect response and 1 being the correct response. The error bars for the predicted data represent the 95% credible interval.*


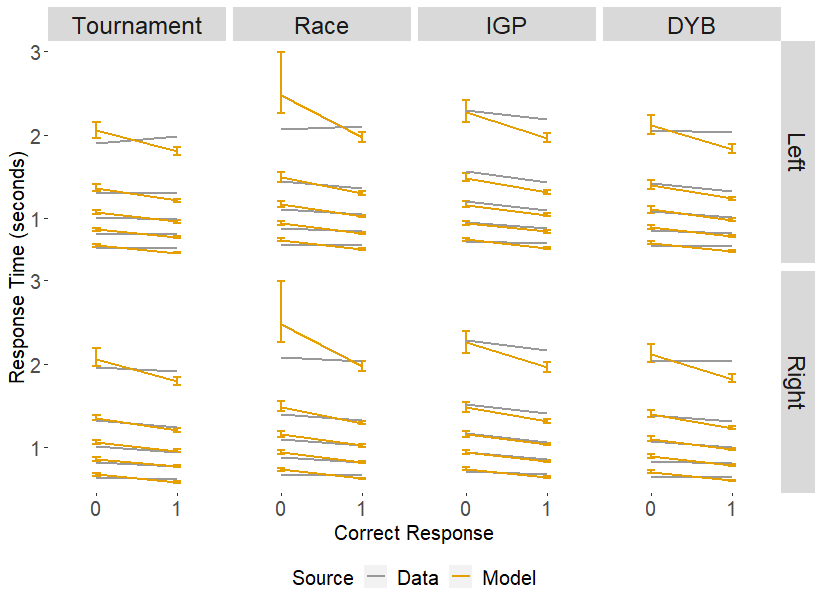


Parameter recovery analysis was conducted to determine whether the estimated parameters were recoverable. Simulated data, with the same number of trials as the real data, were generated using each participants’ mean value in each condition for each parameter. This allowed for comparison between the data generating parameters and the estimated parameters. Figures 7 and 8 below, show the data-generating and recovered parameters graphed against each other. Figure 7 shows the threshold parameters for each condition and Figure 8 shows the difference between drift rate parameters for each condition. Based on this analysis, the recovery of the parameters was good, meaning the parameters are well estimated given the sample size and design.

**Figure 7**

*Actual and recovered values for the mean threshold parameter for each condition.*


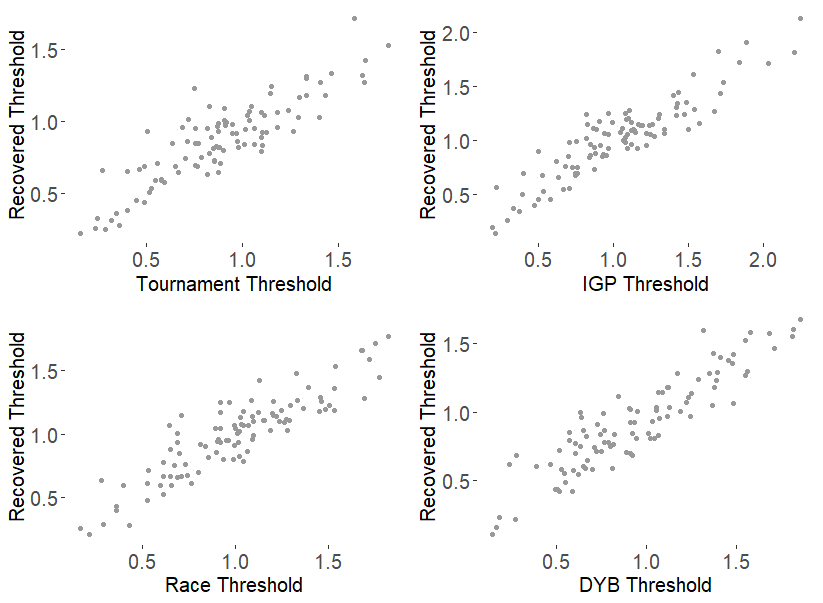


**Figure 8**

*Actual and recovered values for the mean difference between drift rates parameter for each condition.*


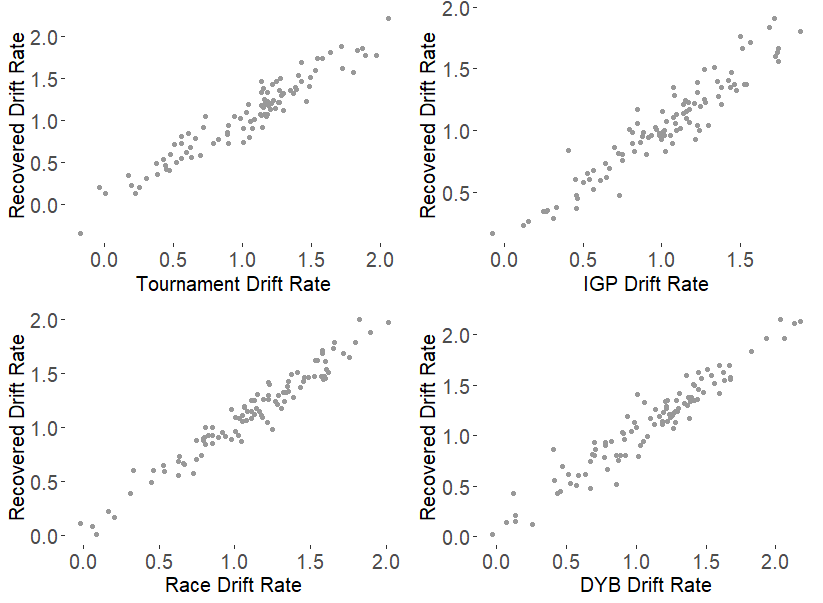


**Relationship Between Threshold and Performance**

In order to examine the relationship between threshold and performance in this task, we simulated the competitions in our experiment using a range of different threshold values. The experiment was simulated (with 1000 samples for each episode) using real participant parameter estimates, except for threshold which was set at a specific value. We ran thresholds from 0.001 to 0.01 in steps of 0.001, and then from 0.01 to 4.00 in steps of 0.01, totalling 409 thresholds. Below are some graphs that show the thresholds and how they relate to the final score of an episode (Figure 9) and the win rate (Figure 10).

There are multiple ways to conceptualise optimality, and these different ways may only apply to some of the conditions. In terms of score, the optimal way to gain points in the tournament and DYB conditions appears to be setting very low thresholds. It is uncertain whether this pattern holds true for the race and IGP conditions, as their episodes ended when someone reaches eight points, potentially obscuring the relationship between threshold and score. In terms of win rate, there is wider variation as a function of threshold, especially in the competitive conditions. Since tournaments and races are dependent on both the individual’s and the opponent’s score, lower thresholds do not always lead to higher win rates, as the opponent’s performance is equally as important as the individual’s. For the IGP condition, lower thresholds do appear to be associated with higher win rates, though the magnitude of this association seems to be heavily dependent on individual ability. There was no win condition in the DYB condition.

Based on the final score results, lower thresholds do not necessarily lead to better scores, meaning participants do not need to set lower thresholds to perform better. This lines up with our findings of differences in thresholds where, compared to the DYB condition, IGP and Race had higher thresholds (potentially due to their eight-point score limit), while tournament had lower thresholds, as these were associated with better performance.

It's worth noting that the finding that thresholds of approximately zero produce the best performance (in some conditions) does not mean that people perform best in this task when they collect no evidence. The findings mean that people perform best when the threshold is near the upper bound of the starting point of evidence. Even when threshold takes a value of zero, in most cases, the actual starting point will be far below the threshold, particularly when the upper bound of the starting point is high. It would be possible to simulate competitions in which the threshold could be below the starting point of evidence, but this is generally not permitted when estimating parameters using the LBA (and was not permitted in our analyses).

**Figure 9**

*Results of the optimal threshold analysis on average score at the end of a competitive episode, at the individual level and averaged across individuals. Note that in the Individual Goal Pursuit and Race conditions, scores could not go above eight.*


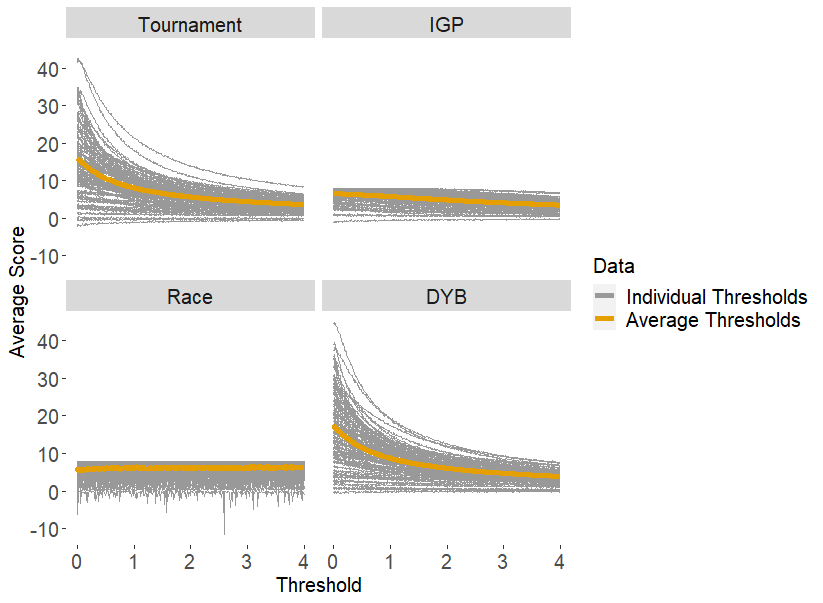


**Figure 10**

*Results of the optimal threshold analysis on average win rate across competitive episodes, at the individual level and averaged across individuals. Note that the in the Do Your Best condition, there was no opponent or target score, meaning there was no metric for “winning.”*


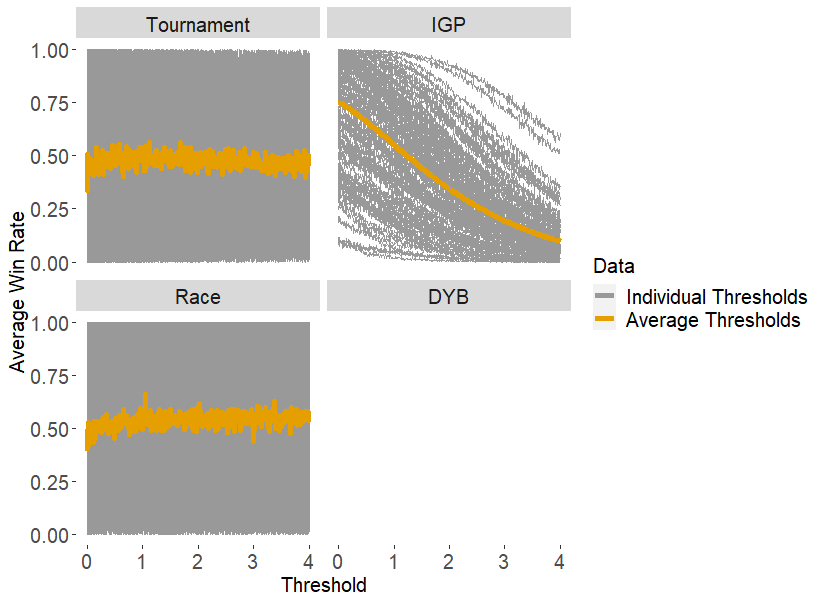


**Posterior Representative Analysis**

To address the effects of competition type at the individual level and see if they are consistent with the overall effects, we have conducted a posterior representative analysis. We randomly sampled 8000 values for an additional participant (without real data) from the truncated group distributions of threshold and drift rate (based on their means and standard deviations) and built a distribution of these values across competition type. These distributions are shown in Figure 11 (for threshold) and Figure 12 (for drift rate). From these distributions, it appears the individual level effects on threshold and drift rate do not contradict

the overall effects across individuals.

**Figure 11**

*Posterior representative analysis of the effect of competition type on threshold.*


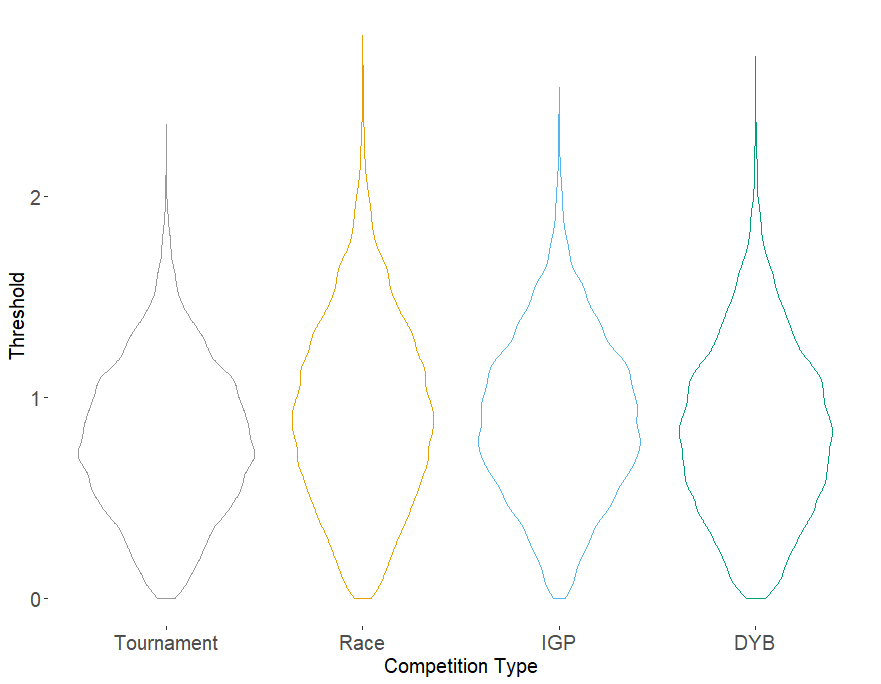


**Figure 12**

*Posterior representative analysis of the effect of competition type on the difference between the drift rates for the correct and incorrect decision.*


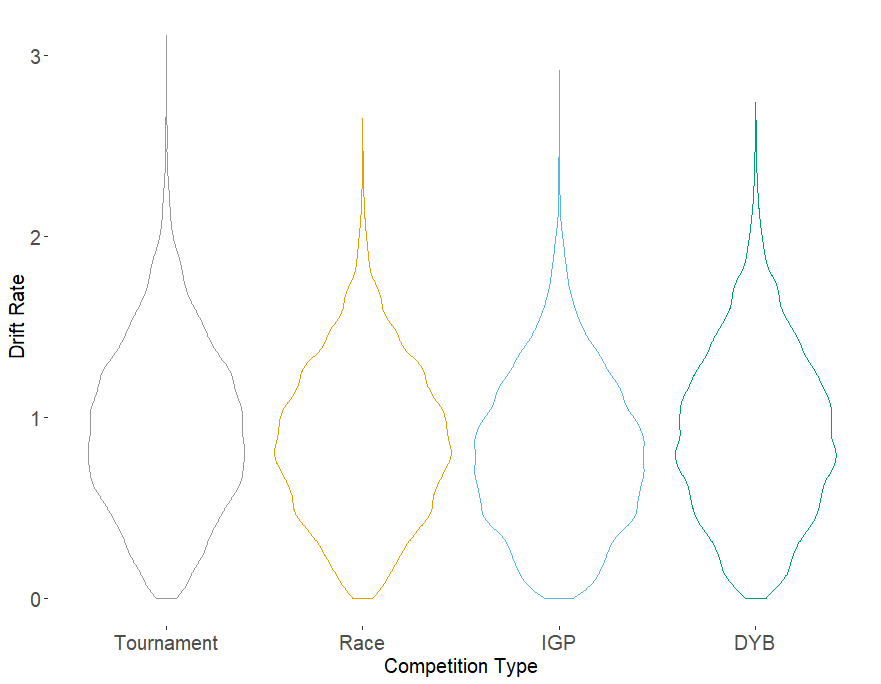

Supplement: Supplementary file 1 — (DOCX 457 kb) [file 13423_2022_2213_MOESM1_ESM.docx]
